# NAC and Vitamin D Restore CNS Glutathione in Endotoxin-Sensitized Neonatal Hypoxic-Ischemic Rats

**DOI:** 10.3390/antiox10030489

**Published:** 2021-03-20

**Authors:** Lauren E. Adams, Hunter G. Moss, Danielle W. Lowe, Truman Brown, Donald B. Wiest, Bruce W. Hollis, Inderjit Singh, Dorothea D. Jenkins

**Affiliations:** 1 Department of Pediatrics, 10 McLellan Banks Dr, Medical University of South Carolina, Charleston, SC 29425, USA; adamsla@musc.edu (L.E.A.); hollisb@musc.edu (B.W.H.); singhi@musc.edu (I.S.); 2 Center for Biomedical Imaging, Department of Radiology, Medical University of South Carolina, 68 President St. Room 205, Charleston, SC 29425, USA; mossh@musc.edu (H.G.M.); brotrr@musc.edu (T.B.); 3 Department of Psychiatry, Medical University of South Carolina, 67 Presidents St., MSC 861, Charleston, SC 29425, USA; clarkdw@musc.edu; 4 Department of Pharmacy and Clinical Sciences, College of Pharmacy, Medical University of South Carolina, Charleston, SC 29425, USA; wiestdb@musc.edu

**Keywords:** glutathione, glutamate, oxidative stress, hypoxia ischemia, endotoxin, magnetic resonance spectroscopy, *N*-acetylcysteine, vitamin D

## Abstract

Therapeutic hypothermia does not improve outcomes in neonatal hypoxia ischemia (HI) complicated by perinatal infection, due to well-described, pre-existing oxidative stress and neuroinflammation that shorten the therapeutic window. For effective neuroprotection post-injury, we must first define and then target CNS metabolomic changes immediately after endotoxin-sensitized HI (LPS-HI). We hypothesized that LPS-HI would acutely deplete reduced glutathione (GSH), indicating overwhelming oxidative stress in spite of hypothermia treatment in neonatal rats. Post-natal day 7 rats were randomized to sham ligation, or severe LPS-HI (0.5 mg/kg 4 h before right carotid artery ligation, 90 min 8% O_2_), followed by hypothermia alone or with *N*-acetylcysteine (25 mg/kg) and vitamin D (1,25(OH)_2_D_3_, 0.05 μg/kg) (NVD). We quantified in vivo CNS metabolites by serial 7T MR Spectroscopy before, immediately after LPS-HI, and after treatment, along with terminal plasma drug concentrations. GSH was significantly decreased in all LPS-HI rats compared with baseline and sham controls. Two hours of hypothermia alone did not improve GSH and allowed glutamate + glutamine (GLX) to increase. Within 1 h of administration, NVD increased GSH close to baseline and suppressed GLX. The combination of NVD with hypothermia rapidly improved cellular redox status after LPS-HI, potentially inhibiting important secondary injury cascades and allowing more time for hypothermic neuroprotection.

## 1. Introduction

Intrauterine inflammation and infection induce fetal inflammation, sensitize the fetal brain to secondary hypoxic ischemic (HI) injury, and increase the severity of HI brain injury [1,2,3,4]. Chorioamnionitis and/or funisitis are present in the placentas of approximately 30% of infants with hypoxic ischemic encephalopathy (HIE) [5]. Hypothermia treatment is the standard of care in moderate to severe HIE, including neonates exposed to intrauterine inflammation. While therapeutic hypothermia provides effective neuroprotection in both animals and neonates in uncomplicated HI, there is inconsistent benefit when infection/inflammation precedes HI [6,7,8,9]. As perinatal infection may not be identified prior to delivery [10], adjuvant postnatal therapies that improve on hypothermia’s neuroprotection should also address the combined injury of inflammation and HI [5].

Few therapies tested in preclinical HI or lipopolysaccharide-sensitized HI (LPS-HI) models show synergetic effects with hypothermia [11,12,13,14,15,16]. While this may be due to the fact that therapeutic hypothermia already encompasses multiple mechanisms of neuroprotection, it may also be the case that pre-existing neuroinflammation requires much more rapid and targeted treatment of specific vulnerabilities [8], such as reduced glutathione (GSH) depletion. Fetal neuroinflammation in utero activates toll-like receptors, pro-inflammatory cytokines and oxidative stress, decreasing cellular reserves prior to HI [17,18] and leaving the neonate unable to counteract further hypoxic ischemic challenges at birth [19]. If oxidative stress and neuroinflammatory cascades are already activated before HI, the therapeutic window before secondary ATP depletion and irreversible injury is much shorter than in uncomplicated HI. In a normothermic neonatal rat, LPS-HI decreases GSH as soon as 2 h after HI [17]. What is not known is how rapidly CNS metabolomics are affected by LPS-HI and if hypothermia or other targeted treatments can act quickly enough within this 2 h time period to change the pro-oxidant CNS milieu in order to mitigate secondary injury.

Neutralizing oxidative stress early after injury is an important step in neuroprotective strategies. Rescuing GSH is necessary and sufficient for neuroprotection in glutamate toxicity, stroke and other injury models [20,21,22,23,24,25,26,27]. Antioxidant treatment with *N*-acetylcysteine (NAC) scavenges oxidative free radicals and provides cysteine, a critical precursor for GSH synthesis. In an LPS-HI neonatal model, NAC at 200 mg/kg increased GSH when given before and after HI under normothermic conditions [17]. While prior treatment with NAC is neuroprotective in normothermic neonatal LPS models, we have not determined whether NAC can act quickly when administered after LPS-HI to counteract the abnormalities of pre-existing neuroinflammation and rescue GSH.

We therefore designed these experiments to determine the hyperacute effects of LPS-HI injury on CNS metabolomics, and if treatment with therapeutic hypothermia alone or with a combination of antioxidant therapies could mitigate these during a critical early stage of secondary injury. We used serial magnetic resonance spectroscopy (MRS) measurements in the same animals before and after injury to analyze responses within individual animals, and to be able to draw conclusions in spite of the significant heterogeneity inherent in neonatal HI models, which is also highly relevant to the clinical disease process. We hypothesized that LPS-HI injury would rapidly decrease GSH in the ipsilateral hemisphere, and that hypothermia treatment alone would not mitigate this oxidative stress.

We tested the combination of NAC and 1,25(OH)_2_D (NVD) with hypothermia, as we have previously shown that they are neuroprotective postnatally in both sexes in a neonatal model of severe HI [28]. NAC and 1,25(OH)_2_D may act synergistically to increase the intracellular antioxidant capacity and decrease inflammation: NAC provides the rate-limiting glutathione substrate while vitamin D induces GSH synthetic enzymes cysteine-glutamate ligase and glutathione reductase [29,30]. By augmenting both aspects of GSH synthesis, we postulated that NVD treatment might be more effective at replenishing CNS GSH rapidly in the early phase of LPS-HI injury than hypothermia alone. We related these CNS metabolite changes to plasma concentrations of NAC and 1,25(OH)_2_D for translational evidence that NVD crosses the blood–brain barrier quickly with significant therapeutic effects on CNS metabolomics, facilitating hypothermia’s neuroprotective effects.

## 2. Materials and Methods

### 2.1. Validation of GSH by MRS

**Figure 1:**
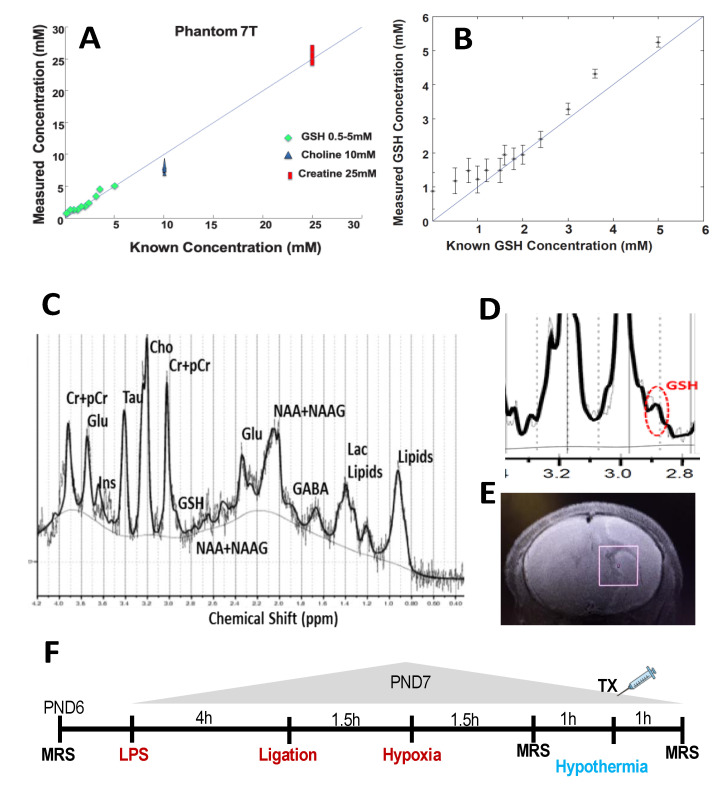
(**A**) Standard curve of LCModel quantification of metabolites (reduced glutathione (GSH), total choline (tCho) and total creatine (tCr), mM) using our specialized basis set, versus known concentrations in phantom samples in the Bruker 7T MRI. (**B**) Expanded view of GSH standard curve using LCModel quantification in phantom samples. (**C**) Representative rat brain spectra with major metabolites labeled (7T Bruker MRI). (**D**) Cysteinyl residue of reduced glutathione gives a single peak at 2.95 ppm. (**E**) Voxel box positioning in the right hemisphere, representative image. (**F**) Study timeline (MRS scan times noted; TX represents NVD or saline dosing).

Prior to animal experimentation, we used VeSPA [31], a spectral simulation program, to create a custom simulated echo acquisition mode (STEAM) basis set for use at echo time (TE) 3 ms on 7 Tesla MRI that included GSH and all standard metabolites of total choline (tCho, choline + phosphocholine), total creatine (tCr, creatin + phosphocreatine), total *N*-Acetylaspartate (NAA, *N*-Acetylaspartate + *N*-acetylaspartylglutamate), glutamate (Glu), glutamine (Gln), glutamate + glutamine (GLX), taurine (Tau) and inositol (Ins). The basis set was calibrated and then imported into the LCModel for automatic spectral fitting of the data [32]. Phantom solutions (0.5–5 mM GSH, 5 mM dithiothreitol, 10 mM choline, 25 mM creatine, phosphate buffered saline, pH 7.1) were used to validate GSH, creatine and choline quantification by MRS, using the water peak as a standard. The quantification of GSH and tCr by the LCModel processing of MRS spectra with our specialized basis set showed excellent correlation with known concentrations of these metabolites in the phantom solution (Figure 1A,B).

### 2.2. Animals and Reagents

Postnatal day seven (PND 7) Sprague Dawley rats were used for all experiments (Harlan, Indianapolis, IN, USA). Litters were culled to 6 pups per sex on PND 2. Animals were housed in the animal care facility of the Medical University of South Carolina (MUSC) and were kept in a 12/12 h light/dark cycle with standard chow and water ad libitum. All procedures were in accordance with the approved protocol #1678 by MUSC’s Institutional Animal Care and Use Committee and the Guide for the Care and Use of Laboratory Animals adopted by the National Institutes of Health and approved by the MUSC Animal Care and Use Committee. Animal reagents used for this study included: lipopolysaccharide from E. coli O55:B5 (LPS #L5418, Sigma, St. Louis, MO, USA); Novaplus™ (Isoflurane, USP, Primal Healthcare, Andhra Pradesh, India); pharmaceutical grade *N*-Acetylcysteine (Acetadote, Cumberland Pharmaceuticals, Nashville, TN, USA) and pharmaceutical-grade calcitriol, 1,25(OH)_2_D_3_ (Calcijex^®^, Abbott Laboratories, North Chicago, IL, USA).

### 2.3. Endotoxin Sensitized Hypoxic Ischemic Injury Animal Model

For this severe model of LPS-HI injury, LPS 0.5 mg/kg in saline was administered by intraperitoneal injection 4 h prior [18] to right carotid artery ligation under isoflurane anesthesia and 21% oxygen, followed by 1.5 h rest, then 1.5 h exposure to 8% O_2_. This hypoxia exposure is considerably longer than the 50 min used in other moderate endotoxin-sensitized HI neonatal models [9,18]. LPS-HI pups were then separated in a temperature-controlled chamber maintained at 30 ± 0.5 °C for 2 h for hypothermia treatment. Animals received only 21% oxygen for resuscitation, if necessary. Sham-operated animals received saline injections, underwent anesthesia and right carotid artery isolation, but no ligation, hypoxia, or hypothermia.

### 2.4. Experimental Design

Sixty-six PND 6 male and female rats were block randomized within the litter in a 1:4 allocation to sham or LPS-HI prior to baseline MRS scans. Twelve hours after baseline MRS, on PND 7, the sham group received sham surgery and saline injection, and LPS-HI animals were subjected to LPS-HI injury. The surviving LPS-HI animals were then stratified by sex and randomly assigned to receive hypothermia and an equal volume of saline (VEH) or a single treatment (NVD) of NAC (Acetadote 25 mg/kg) and 1,25(OH)_2_D_3_ (calcitriol 0.05 μg/kg). Rat pups were removed for intraperitoneal (i.p.) injection 1 h after initiation of hypothermia, then quickly replaced in the hypothermia chamber. Stratification by sex within the litter assured as much as possible that equal numbers of male and female animals were randomized to each group. To accommodate the repeated 20 min MRS scan, we staggered start times for each animal’s LPS/saline injection, surgery and hypothermia treatment. We were able to study only one litter per day due to the 16 h protocol. Pups were euthanized with isoflurane anesthesia and decapitation according to the Institutional Animal Care and Use Committee protocol. Sample size power analysis: From reports that employed biochemical GSH assays [17], we estimated LPS-HI would result in a 30% decrease in MRS GSH compared with baseline, and NVD would result in an increase in GSH to 80% of pre-LPS-HI concentrations, yielding a sample size of *n* = 12/group, with 80% power, a = 0.05.

### 2.5. Magnetic Resonance Imaging and Spectroscopy

MRI was performed in the supine position in an adapted holder under continuous anesthesia with 0.5–1.5% isoflurane in 21% oxygen, in a small animal 7 Tesla Bruker Biospec 70/30 scanner (Bruker Biospin, Germany) with a 12 cm gradient and shim coil set (B-GA 12S2). LPS-treated animals required much less isoflurane for anesthesia than sham animals. Standard T2 scans were performed for anatomical positioning of the voxel (Figure 1E). After reconstruction and voxel placement, we acquired an MRS water reference scan for each animal, followed by STEAM sequence (TE = 3 ms, TR 1500 ms, TM = 10 ms, number of averages = 512/1024, Voxel Size = 3 × 3 × 3 mm^3^) in the right hemisphere (Figure 1C–E). Three MRS scans were obtained on LPS-HI animals: a baseline pre-LPS-HI scan on PND 6 (PRE); a post-LPS-HI scan on PND7 immediately after hypoxia (POST HI); and a post-HYPO scan immediately after hypothermia (1 h after saline or NVD treatment). Sham animals underwent 2 MRS scans, on PND 6 and PND 7 after sham surgery. Respiratory monitoring and temperature servo control (36.5 °C) was ensured throughout the procedure. The study timeline is shown in Figure 1F.

### 2.6. MRS Data Processing

LCModel fitting of the spectra, evaluation of spectral quality, and quantification of metabolites were performed by a researcher blinded to the treatment group. To ensure good quality scans, the inclusion criteria for processed MRS were based on spectral quality as reported by the LCModel (full width at half maximum ≥ 0.1 ppm, signal to noise ratio ≥ 5) [33] as well as for obvious artifacts due to gross motion and poor water suppression. Using explicit formulas for LCModel, a single water attenuation coefficient was calculated and implemented when processing all spectra. No partial volume correction was taken into account with these immature animals and we assumed brain matter uniformity. The following metabolites were analyzed for absolute concentrations in the right hemisphere: GSH, GLX, tCr, NAA, tCho, Tau, Ins and LAC. Major peaks for these metabolites are noted on a representative spectrum (Figure 1C,D). The inclusion of metabolite concentrations was based on Cramer Rao < 15% for all metabolites, excluding lactate. The range of Cramer Rao bounds for LAC is given in the results.

### 2.7. NAC and Vitamin D Assays

Blood samples were drawn immediately after the final scan by cardiac puncture at time of euthanasia, approximately 1.5 h after dosing saline or NVD, and analyzed for NAC and Vitamin D plasma levels. If an animal died prior to the completion of hypothermia, we did not obtain blood for drug concentrations. Blood samples (0.5 mL) were collected in sodium EDTA tubes, then immediately centrifuged and plasma stored in polypropylene tubes at −80 °C until analysis.

Total NAC plasma concentrations (i.e., oxidized, reduced and protein-bound drug) were determined using a modified, reverse-phase, high-performance liquid chromatography method with penicillamine as the internal standard, as previously described [34]. Plasma samples were initially treated with dithiothreitol to reduce available oxidized NAC (NAC2) and then derivatized with *N*-(1-pyrenyl)maleimide (NPM). The NAC–NPM adduct was then analyzed by fluorescence detection. The limit of sensitivity for the assay was 6.0 µmol/L. The five-point standard curve was linear and reproducible over the range of 60 to 3000 µmol/L (*R*^2^ > 0.99). The coefficients of variation for the within-run and between-run precision were all less than 10%.

25(OH)D and 1,25(OH)2D levels were measured using a rapid, direct radioimmunoassay in Dr. Hollis’ laboratory as previously described, with a lower limit of detection of 2 ng/mL for 25(OH)D and 15 pg/mL for 1,25(OH)_2_D [35,36].

### 2.8. Statistical Analysis

We used generalized linear mixed models (GLMMs) in our two primary analyses. For maximum power to detect differences due to injury, we compared rats for metabolite changes between sham- and LPS-HI-exposed rats at baseline, within the sham group from pre-PND 6 to post-sham surgery PND 7 scans, and within the LPS-HI rats from before (PRE) to after injury (POST HI, before treatment). All animals in the LPS-HI saline and NVD groups were treated the same through injury and survivors were randomized after POST-HI scan stratified by sex, and thus were analyzed as a group for LPS-HI-induced changes in metabolite concentrations over the first two scans (*scan* and *LPS-HI* as main effects, *sex* as covariate). Secondly, we constructed a model to determine the treatment effect over time, and any influence of scan time point and sex on metabolite concentrations, using *scan time, scan-within-treatment*, sex*treatment and sex* treatment*scan as fixed effects, and *pup ID* as random effects. Including the interaction factor *scan-within-treatment* to test for within-group differences, this gave the best model fit by Akaike-corrected information criteria (AIC). Pairwise analyses were corrected for multiple comparisons by sequential Bonferroni adjustments. We used chi-squared or fishers exact tests for numbers of animals that had detectable LAC or responded to treatment. Spearman’s rho was used for non-parametric analysis of plasma NAC. Pearson’s was used for all other correlations. All statistical calculations were performed using SPSS^®^ vs. 25 (IBM®, Armonk, NY, USA), with significance designated as *p* < 0.05 after Bonferroni adjustments.

## 3. Results

### 3.1. LPS-HI Morbidity and Mortality

**Figure 2:**
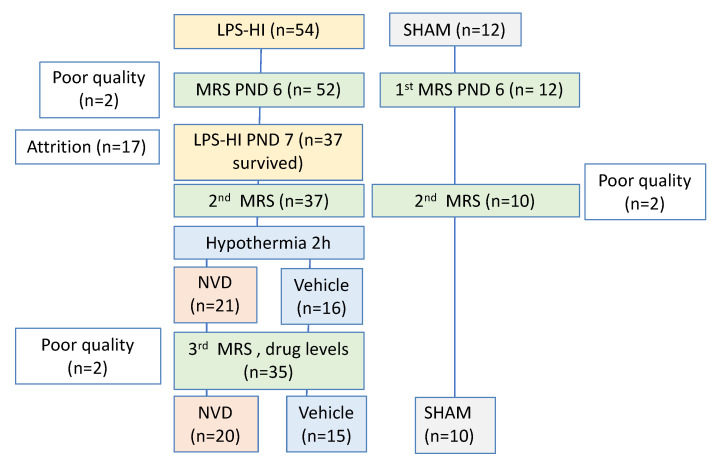
Overview Study Flow Diagram.

We randomized 66 rats (Figure 2) from 10 litters, and 17 rats died during or after the LPS-HI injury, before the third scan (10 males, 7 females). We obtained (1) pre-injury, baseline scans on PND 6 for 54 LPS-HI rats and 12 sham rats; (2) POST HI scans on 37 LPS-HI rats and 10 sham animals; and (3) POST HYPO scans on 20 NVD and 15 VEH animals. By sex, 23 females (*n* = 9 NVD, *n* = 6 VEH, *n* = 6 sham) and 21 males (*n* = 11 NVD, *n* = 9 VEH, *n* = 4 sham) survived to the final time point, with scans of sufficient quality for analysis. In total we analyzed 146 MRS scans (22 sham, 59 VEH and 65 NVD scans). The plasma volume was adequate for the determination of 1,25(OH)_2_D and 25(OH)D concentrations by radio-immunoassay in 20 animals (*n* = 12 LPS-HI, 8 sham) and NAC concentrations by HPLC in 13 animals (*n* = 10 NVD, 2 VEH, 1 sham).

### 3.2. LPS-HI Acutely Decreases GSH, GLX and tCho

**Figure 3:**
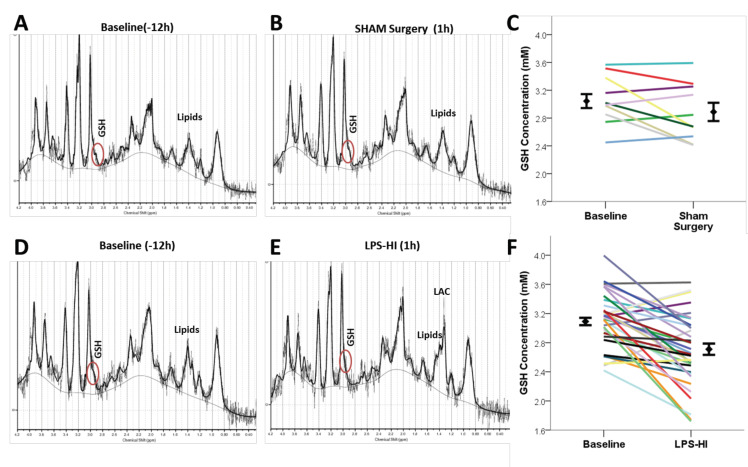
Representative spectra and change in GSH over time from sham (**A**–**C**) and LPS-HI rat pups (**D**–**F**). Lactate peaks are minimal in the sham animals before (**A**) and after sham surgery (**B**) and cannot be distinguished from lipid peaks in the MRS spectra. (**C**) Sham animals show no significant change in GSH before and after sham surgery (*n* = 10). (**D**,**E** ) Lactate peak increases significantly after LPS-HI injury and is clearly visible in spectra from a representative rat (**E**). (**F**) LPS-HI animals show a significant decrease in GSH concentrations after LPS-HI injury (*p* < 0.00017, *n* = 37). Group means and standard errors are noted. Table 1. LPS-HI effects on MRS metabolite concentrations (mM, mean, SE) from PRE (PND 6) to POST-HI (PND 7 sham, or after LPS-HI). *p* values are noted for pairwise comparisons by scan time within LPS-HI group by generalized linear mixed model (GLMM) with Bonferroni adjustments, controlling for sex. The number of adequate spectra for each metabolite varies depending on exclusion due to Cramer Rao bounds >15%.

Representative spectra from a sham and an LPS-HI animal are presented at baseline (Figure 3A,D) and after sham surgery or LPS-HI injury (Figure 3B,E). LPS-HI resulted in a decrease in ipsilateral GSH by estimated mean SE ΔGSH −0.40 ± 0.07 mM from PRE (*n* = 52) to immediately after hypoxia (POST HI, *n* = 37 in all LPS-HI-exposed animals (F = 17.9, *p* < 0.0001, Figure 3F, Table 1). Mean glutamate and glutamine (GLX) decreased significantly after LPS-HI (ΔGLX −1.41 ± 0.15 mM, F = 54, *p* < 0.0001), as did total choline (ΔtCho −0.19 ± 0.05 mM, F = 9, *p* < 0.0001; Table 1). NAA, Tau, and Ins concentrations showed no significant difference between scans.

Sham animals showed no significant change in any metabolite after receiving the same anesthesia, surgery and scan times as LPS-HI rats. There was no difference at baseline scan (PRE) between sham and LPS-HI rats for any metabolite (Table 1).

### 3.3. NVD Improves GSH after LPS-HI Compared with HYPO Alone

**Figure 4:**
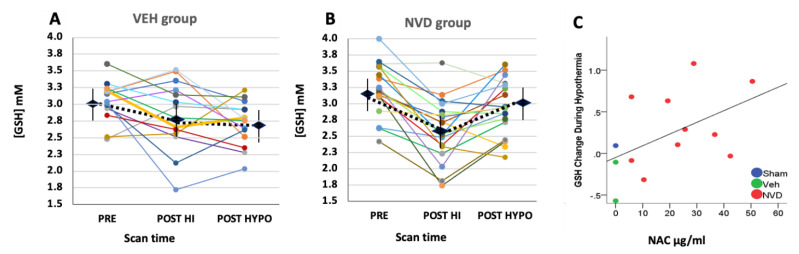
GSH concentrations in individual LPS-HI rats with adequate spectra for all 3 scans by VEH (**A**) or NVD (**B**) treatment over time and compared with NAC plasma concentrations (**C**). (**A**,**B**) Individual changes in GSH over serial scans at baseline (PRE), after LPS-HI (POST HI), and again immediately after 2 h of hypothermia (POST HYPO) in rats with VEH (**A**) or NVD (**B**) treatment. The markers show group mean and SE for each scan time. (**A**) The majority of VEH animals treated with saline do not show significant recovery with hypothermia alone and mean GSH continues to decrease at the third scan (*n* = 14). (**B**) In contrast, NVD treatment results in significant increases in mean GSH with hypothermia (*n* = 18). (**C**) Individual plasma NAC concentrations (*n* = 13) sampled immediately after last scan (POST HYPO) versus the change in GSH from immediately before and after NVD/saline. Sham concentration is included for reference.

In the VEH group that received HYPO alone, GSH decreased further from POST HI to POST HYPO and was significantly lower than PRE after hypothermia alone (overall model VEH *n* = 15, F = 5.2, *p* = 0.007, Figure 4A, Table 2). In contrast, GSH improved significantly after NVD at the POST HYPO scan compared with POST HI and was not significantly different from PRE GSH (overall model *n* = 20, F = 17.1, *p* = 0.001; Figure 4B, Table 2). The change in GSH from POST HI to POST HYPO was significantly less with hypothermia alone (ΔGSH VEH −0.08 ± 0.11 mM) than with hypothermia and NVD (ΔGSH +0.32 ± 0.1 mM, *p* = 0.01, Figure 4C). GSH was not significantly different at any time point by sex.

### 3.4. NAC and Active Vitamin D Increase GSH Response Rate in Heterogeneous LPS-HI Injury

VEH animals showed no improvement in mean GSH as a result of saline and hypothermia (*p* = 0.4, Figure 4A, Table 1). However, several individual VEH rats (3/14, 21%, Figure 4A) did respond to hypothermia alone with an increase in GSH. At the same time, NVD increased GSH in 14/18 (78%) rat pups, which is significantly greater than in the VEH group (*p* = 0.004, fisher’s exact test; Figure 4B). The plasma NAC concentrations obtained 1.5 h after i.p. injection of NVD/saline were positively correlated with the change in the CNS GSH from POST HI to POST HYPO (Spearman’s rho = 0.552, *p* = 0.05, Figure 4C).

### 3.5. NAC and Active Vitamin D Suppress Glutamate + Glutamine after LPS-HI Injury

GLX decreased over the three scans in both LPS-HI groups (both F = 27, *p* < 0.0001). However, the VEH group exhibited a significant rebound in GLX after hypothermia alone, primarily due to increased glutamate (ΔGLX +0.76 ± 0.24 mM from PRE, F = 27, *p* = 0.002), whereas NVD continued to suppress glutamate (ΔGLX −0.23 ± 0.21 mM) from the second scan POST HI to the third scan after NVD and hypothermia (Table 2). In NVD rats, tCho decreased significantly with LPS-HI and remained lower at the third scan POST HYPO, while there are no substantive changes in tCho in the VEH group.

**Figure 5:**
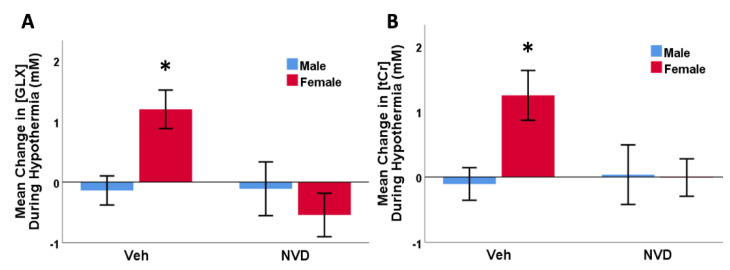
Changes in GLX (**A**) and tCr (**B**) between POST HI and POST HYPO scans by sex within treatment groups. VEH female animals (*n* = 6) showed a significant increase in mean GLX ((**A**), * *p* = 0.007), and Cr ((**B**), * *p* = 0.044) at 2.5 h after injury compared to VEH males (*n* = 9). There was no difference between females (*n* = 9) and males (*n* = 11) in the NVD group.

We found significant differences by sex in response to hypothermia treatment in GLX (F = 46.6, *p* = 0.01) and tCr (F = 4.5, *p* = 0.013). VEH females showed a rebound in CNS GLX and tCr concentrations, with significantly higher GLX (ΔGLX +0.89 ± 0.34 mM) and tCr (ΔtCr +0.76 ± 0.30 mM) after hypothermia treatment alone than males (Figure 5). NVD equalized this difference between males and females, and neither GLX nor tCr were significantly different between sexes at either POST HI or POST HYPO scans.

### 3.6. Lactate Increases after LPS-HI Injury and Persists after HYPO

Lactate was undetectable on PRE scans, but we observed significantly increased lactate peaks in the spectra from 22 of 37 animals (60%) obtained immediately after LPS-HI injury (Figure 2E). Lactate was quantifiable in POST HI scans with a mean SE of 4.34 ± 0.26 mM, and it persisted in 32% of pups in the POST HYPO period with mean SE 3.12 ± 0.26 mM. There was no difference in the numbers of animals that had detectable lactate in either NVD or VEH groups by chi-squared analysis. Spectral fitting yielded Cramer Rao standard deviations of 7–38%, consistent with greater difficulties fitting the lactate peak within overlapping lipid residues.

### 3.7. LPS-HI Injury Decreases Plasma 1,25(OH)_2_D

**Figure 6:**
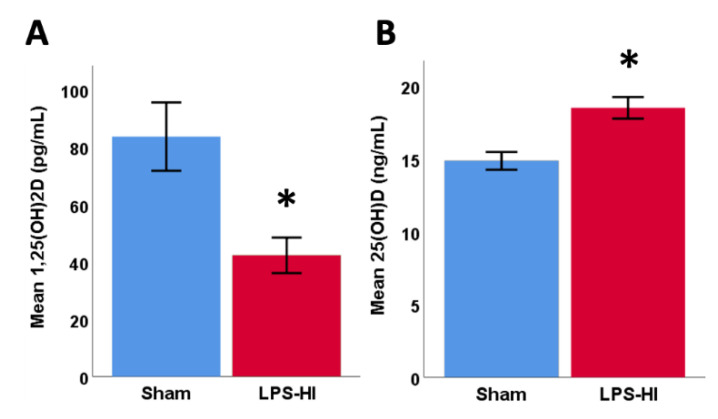
(**A**) Endotoxin-sensitized HI (LPS-HI) animals (*n* = 11), regardless of treatment, show a 50% lower plasma concentration of 1,25(OH)_2_D after LPS-HI than sham animals (*n* = 8, * *p* = 0.004, *t*-test), (**B**) and marginally higher plasma 25(OH)D (+4 ng/mL, *n* = 12) than sham (*n* = 8, * *p* = 0.002, *t*-test).

LPS-HI was associated with a 50% decrease in mean circulating 1,25(OH)_2_D concentrations (42 ± 21 pg/mL) compared with sham animals (84 ± 34 pg/mL, Figure 6A) in blood collected 2.5 h after hypoxia. Neither hypothermia alone nor NVD in combination with hypothermia restored plasma concentrations of active hormone 1,25(OH)_2_D within this time period (1.5 h after NVD treatment). At the same time, all LPS-HI animals had slightly higher circulating 25(OH)D_3_ (18.5 ± 2.6 ng/mL) than sham rats (14.9 ± 1.7 ng/mL, Figure 6B), indicating the increased utilization or degradation of vitamin D, and attempts to mobilize the inactive precursor to maintain active hormone levels. There were no differences by sex within or between the sham and LPS-HI groups.

### 3.8. T2 Evidence of Infarcts

**Figure 7:**
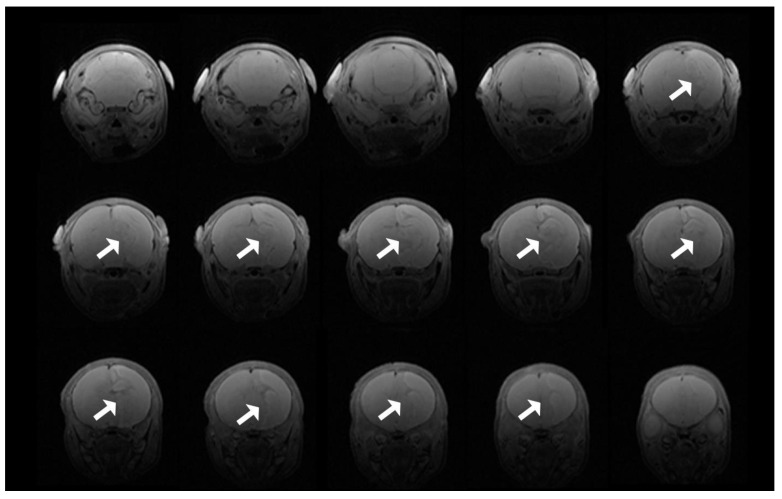
T2 scan showing progression of infarction for an animal from the VEH group. Of note, the right hemisphere is on the left side of the magnetic resonance imaging (MRI) images.

T2 images demonstrated hyperacute infarction in four animals after hypothermia treatment, two in each of the VEH and NVD groups (Figure 7) within 2 h of hypoxia, which usually evolves over 24 h [37].

## 4. Discussion

Using serial in vivo 7T MRS, we show that endotoxin-sensitized HI injury significantly and immediately changes CNS redox status in a neonatal model, decreasing the intracellular antioxidant concentrations of reduced glutathione GSH in the affected hemisphere immediately after hypoxia, compared with pre-injury and with sham controls. The clinical standard of care for HIE, hypothermia, did not rescue mean GSH within 2 h and concentrations continued to decrease in the POST HYPO period. However, one dose of *N*-acetylcysteine (25 mg/kg) and calcitriol (0.05 mcg/kg), administered after 1 h of hypothermia, restored GSH in the affected hemisphere to near pre-injury levels POST HYPO in the NVD group. Further, 78% of NVD-treated rat pups showed a significant increase in GSH, while only 21% of rat pups treated with hypothermia alone had increased GSH. Plasma NAC positively correlated with CNS GSH, indicating that NAC rapidly crossed the blood–brain barrier to increase GSH [38].

Previous work in an LPS-HI neonatal rat model using biochemical assays of GSH agree with our in vivo MRS findings. In one investigation in neonatal rats, LPS prior to HI increased the depletion of GSH and increased F2-isoprostanes and peroxynitrite derivatives (nitrosylated proteins) at 2 h after HI compared with controls and LPS alone [17]. NAC treatment at 200 mg/kg prior to or immediately after LPS-HI provided neuroprotection, but not 25 mg/kg or when NAC administration was delayed after injury under normothermic conditions [17]. We tested NVD under hypothermic conditions, as hypothermia is now the standard of care for hypoxic ischemic injury in human neonates. In our severe HI and now LPS-HI neonatal rat models, we have shown that low-dose NAC (25 mg/kg) combined with active vitamin D and hypothermia improves neuroinflammation and provides neuroprotection (HI) [28], as well as facilitating facilitates increased glutathione and decreased glutamate (LPS-HI) over hypothermia alone. Taken together, the data suggest that hypothermia decreases the dose of NAC required to affect redox state and outcomes [17,28,39].

Reduced glutathione plays a central role in cell survival in acute HI and neuroinflammation and is an important outcome measure [40,41,42,43,44]. GSH is oxidized to the dimer GSSG by glutathione peroxidase after detoxifying superoxide and peroxynitrite free radicals [45]. The depletion of GSH triggers or potentiates glutamate- and nitric oxide-induced apoptosis [26,46,47]. If oxidative stress and GSH depletion continue, secondary ATP depletion and glutamate-induced cell death follows the initial repletion of ATP [20]. Restoring GSH, particularly in the mitochondria of astrocytes, is necessary and sufficient for neuroprotection from neuronal glutamate toxicity [20], and is essential for cell survival after multiple different insults [21,22,23,24,27,45,48,49].

NVD also mitigates the increase in GLX seen in the hypothermia alone LPS-HI rat pups. While resolving glutamate and glutamine peaks with confidence is controversial in MR spectroscopy, glutamate accounts for the majority of the GLX peak complex and GLX concentration. The depletion of GSH been shown to augment glutamate toxicity after HI, and increased extracellular glutamate inhibits cystine uptake and leads to further GSH depletion [48]. We found an increase in GLX and GSH depletion after hypothermia alone, supporting the relationship of lower GSH and increased glutamate toxicity with neuroinflammation in this severe LPS-HI model, as found by other investigators after HI alone [48]. Conversely, NVD resulted in an increase in GSH and decrease in glutamate concentrations, measured by GLX. Surprisingly, the increase in glutamate was seen primarily in the female rats in the vehicle group, rather than in males. Few reports have addressed glutamate toxicity as a sexually dimorphic injury pathway, but one such study showed that female PND7 rats had greater hippocampal injury than males after administration of the glutamate receptor agonist kainate [50]. However, males and females may have different time courses of glutamate, and our time point may have been too early to observe an increase in males.

The neurohormone 1,25(OH)_2_D plays an important role in neuroprotection in animal models [28,51,52,53,54,55,56], as well as in human stroke and other CNS diseases [35,57,58,59,60,61,62,63,64]. Studies also indicate that inflammation results in the degradation of active vitamin D that may persist for weeks after injury [28,65]. At 11 days after severe HI in neonatal rats, inducible nitric oxide synthase expression in the CNS correlated strongly with the expression of the CYP27B1 enzymes responsible for vitamin D catabolism [28]. Here we report that active vitamin D is dramatically decreased in neonatal rat plasma 2.5 h after LPS-HI, much more quickly than previously demonstrated. These data add to our and others’ work showing vitamin D deficiency and increased vitamin D degradation in neuroinflammatory conditions: in human HIE neonates [35,66], in animal models of HI [28], after antenatal endotoxin exposure [65], and in other conditions of immune activation [36]. These data indicate that vitamin D metabolism is disrupted in both HI and intrauterine inflammation.

Perhaps because of increased vitamin D catabolism with inflammation, we did not demonstrate an increase in plasma 1,25(OH)_2_D concentration after the administration of calcitriol in LPS-HI, unlike a previous report in normal adult rats, in which serum 1,25(OH)_2_D was increased one hour after i.p. administration [67]. This may be due to its rapid dispersal into tissues and increased utilization, or the metabolism of active hormones in inflammatory conditions [28,35]. In HIE infants, vitamin D-binding proteins and albumin that normally function to maintain circulating vitamin D concentrations and limit distribution into tissues are significantly decreased, even with hypothermia [35].

Active vitamin D is a neurohormone that regulates the transcription of multiple genes, including the GSH synthetic enzymes that are inhibited by acidosis [29,52,56,68]. In our work, NAC and active vitamin D acutely and significantly increase GSH in LPS-HI rats and in the basal ganglia on PND 5 after rewarming from hypothermia treatment, within 12–30 min after completing the infusion in HIE neonates [38]. Using biochemical assays, other investigations determined that NAC and active vitamin D increase GSH [29]. Similar to our strategy of using NVD combination therapy to increase intracellular GSH, other investigators have paired NAC with probencid in the treatment of pediatric traumatic brain injury to increase GSH synthesis and inhibit the transport of GSH out of the cell [24,69]. We did not investigate the effect of NVD synergy on GSH synthesis with our targeted study design in this proof-of-concept study. Improved intracellular redox status has been shown to augment 1,25(OH)_2_D binding to its receptor in animal models, enhancing multiple vitamin D signaling transduction pathways [70]. It is also possible that the co-administration of NAC and vitamin D have synergistic effects on important anti-inflammatory and anti-apoptotic pathways other than GSH synthesis.

The use of MRS for measuring hyperacute changes in CNS metabolic profiles represents a non-invasive method for assessing the precise evolution of cellular injury [71,72]. The advantages of MRS quantification versus biochemical assays of reduced glutathione include the fact that oxidative stress may be measured serially, before and after injury and treatment in the same animals; antioxidant dosing can show definitive CNS target attainment; and the real-time in vivo measurement is not subject to rapid oxidation during tissue processing, as it is with biochemical assay. MRS is a non-invasive method of measuring GSH concentrations that has been validated using short echo STEAM sequences in human adults and neonates [73], and allows preclinical pharmacokinetic and pharmacodynamic studies to quickly translate effective doses of therapeutics to clinical studies. Newer software programs use MRS pattern recognition to classify infarct evolution in adult stroke models, illustrating the ability of serial metabolomics to discriminate between core and penumbra after HI [73]. In addition, MRS can quantify cellular energetics and redox simultaneously after CNS injury via measurement of GSH and tCr at the same echo time. This is an important feature of MRS, as the restoring of both are key therapeutic targets. While MR diffusion sequences have proven useful for identifying cytotoxic edema after HI injury [37,74,75], MRS offers significantly more nuanced and complex data on cellular state after injury and recovery, enabling CNS pharmacodynamic studies [76]. MRS metabolomics are valuable biomarkers that provide translationally important diagnostic and therapeutic information after stroke, particularly in the hyperacute phase when therapeutic intervention may improve outcomes.

The limitations of our study include that we did not test whether calcitriol alone could augment GSH production over NAC alone in this short time frame, and we did not perform MRS in the contralateral, LPS-hypoxia-only hemisphere, due to time and funding constraints with repeated MR scans on each rat, and the necessity of processing one litter in one day. Finally, GSSG is not measurable by MRS. However, GSH is the active antioxidant. Further, either GSH or GSSG may be consumed by glutathionylation, an important post-translational regulatory mechanism [48]. Therefore, GSSG concentration or GSH/GSSG ratios are perhaps less important given the current understanding that the synthesis of GSH–GSSG is not a closed loop, and the absolute GSH/GSSG ratio has little additional value for determining cellular ability to handle oxidative stress over GSH alone.

## 5. Conclusions

In one of the most severe models of CNS insult in neonates, serial acute MRS demonstrated significantly decreased concentrations of reduced CNS glutathione after LPS-HI, which were not rescued by hypothermia alone. However, low-dose NAC and active vitamin D administered one hour after the initiation of hypothermia resulted in rapid increases in GSH, which correlated with NAC plasma levels. Serial MRS in the hyperacute phase plays a unique role in translational studies by quantifying redox alterations after CNS injury via GSH and measuring a pharmacodynamic CNS marker of NAC and vitamin D effect that is associated with outcome and response to therapy after this severe injury.

## Figures and Tables

**Table 1:** LPS-HI effects on MRS metabolite concentrations (mM, estimated mean, SE) from PRE (PND 6) to POST-HI (PND 7 sham, or after LPS-HI). *p* values are noted for pairwise comparison (*) by scan time within LPS-HI group by GLMM with Bonferroni adjustments, controlling for sex. The number of adequate spectra for each metabolite varies depending on exclusion due to Cramer Rao bounds > 15%. (Sham group: PRE PND 6 *n* = 12, PND 7 *n* = 10; LPS-HI group: PRE PND 6 *n* = 49–52, POST HI *n* = 36–37).

	GSH		GLX		tCr		NAA		tCho		Ins		Tau	
	SHAM	LPS-HI	SHAM	LPS-HI	SHAM	LPS-HI	SHAM	LPS-HI	SHAM	LPS-HI	SHAM	LPS-HI	SHAM	LPS-HI
PRE PND 6	3.06 ± 0.11	3.06 ± 0.19 *	9.08 ± 0.19	9.00 ± 0.39 *	8.64 ± 0.17	8.58 ± 0.22	4.98 ± 0.19	4.75 ± 0.20	3.02 ± 0.07	2.89 ± 0.17	3.20 ± 0.42	3.13 ± 0.98	17.22 ± 0.48	16.70 ± 1.08
PND 7/ POST HI	2.89 ± 0.11	2.65 ± 0.19 *	9.57 ± 0.20	7.59 ± 0.40 *	8.44 ± 0.18	8.41 ± 023	4.80 ± 0.09	5.02 ± 0.10	3.01 ± 0.07	2.71 ± 0.18	2.95 ± 0.57	3.15 ± 0.90	16.25 ± 0.54	16.18 ± 1.6
*p* value	*ns*	<0.0001	*ns*	<0.0001	*ns*	*ns*	*ns*	*ns*	*ns*	<0.001	*ns*	*ns*	*ns*	*ns*

**Table 2:** NVD vs. saline effects on MRS metabolite concentrations (mM, mean, standard error over 3 scans PRE, POST HI and POST HYPO, in the LPS-HI group treated with hypothermia. Significant changes between scan times within treatment groups are indicated by symbols, along with *p* values for the comparisons (* or ^) by GLMM with sequential Bonferroni adjustments, controlling for sex. The number of adequate spectra for each metabolite varies depending on exclusion due to Cramer Rao bounds >15%.

	GSH		GLX		tCr		NAA		tCho		Ins		Tau	
	VEH	NVD	VEH	NVD	VEH	NVD	VEH	NVD	VEH	NVD	VEH	NVD	VEH	NVD
PRE PND 6	2.97 ± 0.22 *^	3.16 ± 0.32 *^	8.94 ± 0.26 *^	9.02 ± 0.74 *^	8.65 ± 0.27	8.52 ± 0.35	4.96 ± 0.12	5.14 ± 0.13	2.82 ± 0.24	2.97 ± 0.25 *^	3.32 ± 0.91	3.16 ± 0.91	16.70 ± 1.08	16.18 ± 1.60
POST HI	2.72 ± 0.22 **^**	2.58 ± 0.32 *	7.41 ± 0.28 *	7.77 ± 0.74 *	8.37± 0.28 *	8.44 ± 0.35	4.74 ± 0.19	4.94 ± 0.16	2.66 ± 0.24	2.75 ± 0.25 *	2.86 ± 0.92	2.85 ± 0.91	15.85 ± 1.10	15.74 ± 1.62
POST HYPO	2.65 ± 0.22 *	2.90 ± 0.32 *^	8.16 ± 0.29 *^	7.54 ± 0.74 **^**	8.96 ± 0.28 *	8.67 ± 0.35	4.94 ± 0.16	5.14 ± 0.13	2.71 ± 0.24	2.72 ± 0.25 **^**	3.42 ± 0.92	2.83 ± 0.91	16.45 ± 1.10	15.59 ± 1.62
*p* value	<0.05	<0.01	<0.005	<0.0001	≤ 0.01	*ns*	*ns*	*ns*	*ns*	<0.005	*ns*	*ns*	*ns*	*ns*

## Data Availability

Data is contained within the article.

## Abbreviations

Cho	total choline (choline + phosphocholine)
tCr	total creatine (creatine + phosphocreatine)
GLMM	generalized linear mixed model
Gln	glutamine
Glu	glutamate
GLX	glutamate + glutamine
GSH	glutathione
GSSG	glutathione disulfide
HI	hypoxic ischemic
HIE	hypoxic ischemic encephalopathy
Ins	inositol
LAC	lactate
LPS-HI	lipopolysaccharide endotoxin-sensitized HI
MRS	magnetic resonance spectroscopy
NAA	total *N*-Acetylaspartate (*N*-Acetylaspartate + *N*-acetylaspartylglutamate)
NAC	*N*-acetylcysteine
NPM	*N*-(1-pyrenyl)maleimide
NVD	NAC and 1,25(OH)_2_D
PND	postnatal day
POST HI	immediately after hypoxia
POST HYPO	immediately after hypothermia
STEAM	simulated echo acquisition mode
Tau	taurine
TE	echo time
VEH	saline
